# Elucidation of bacterial trehalose-degrading trehalase and trehalose phosphorylase: physiological significance and its potential applications

**DOI:** 10.1093/glycob/cwad084

**Published:** 2023-10-17

**Authors:** Prasansah Shrestha, Jayram Karmacharya, So-Ra Han, Jun Hyuck Lee, Tae-Jin Oh

**Affiliations:** Department of Life Sciences and Biochemical Engineering, Graduate School, Sun Moon University, 70 Sunmoon-ro 221beon-gil, Tangjeong-myeon, Asan-si, Chungcheongnam-do, 31460, South Korea; Department of Life Sciences and Biochemical Engineering, Graduate School, Sun Moon University, 70 Sunmoon-ro 221beon-gil, Tangjeong-myeon, Asan-si, Chungcheongnam-do, 31460, South Korea; Department of Life Sciences and Biochemical Engineering, Graduate School, Sun Moon University, 70 Sunmoon-ro 221beon-gil, Tangjeong-myeon, Asan-si, Chungcheongnam-do, 31460, South Korea; Genome-based Bio-IT Convergence Institute, 70 Sunmoon-ro 221beon-gil, Tangjeong-myeon Asan-si, Chungcheongnam-do, 31460, South Korea; Research Unit of Cryogenic Novel Materials, Korea Polar Research Institute, 26 Songdomirae-ro, Yeonsu-gu, Incheon 21990, South Korea; Department of Life Sciences and Biochemical Engineering, Graduate School, Sun Moon University, 70 Sunmoon-ro 221beon-gil, Tangjeong-myeon, Asan-si, Chungcheongnam-do, 31460, South Korea; Genome-based Bio-IT Convergence Institute, 70 Sunmoon-ro 221beon-gil, Tangjeong-myeon Asan-si, Chungcheongnam-do, 31460, South Korea; Department of Pharmaceutical Engineering and Biotechnology, Sun Moon University, 70 Sunmoon-ro 221beon-gil, Tangjeong-myeon, Asan-si, Chungcheongnam-do 31460, South Korea

**Keywords:** CAZyme, glycoside hydrolase, trehalase, trehalose degradation pathways, trehalose phosphorylase

## Abstract

Bacteria possess diverse metabolic and genetic processes, resulting in the inability of certain bacteria to degrade trehalose. However, some bacteria do have the capability to degrade trehalose, utilizing it as a carbon source, and for defense against environmental stress. Trehalose, a disaccharide, serves as a carbon source for many bacteria, including some that are vital for pathogens. The degradation of trehalose is carried out by enzymes like trehalase (EC 3.2.1.28) and trehalose phosphorylase (EC 2.4.1.64/2.4.1.231), which are classified under the glycoside hydrolase families GH37, GH15, and GH65. Numerous studies and reports have explored the physiological functions, recombinant expression, enzymatic characteristics, and potential applications of these enzymes. However, further research is still being conducted to understand their roles in bacteria. This review aims to provide a comprehensive summary of the current understanding of trehalose degradation pathways in various bacteria, focusing on three key areas: (i) identifying different trehalose-degrading enzymes in Gram-positive and Gram-negative bacteria, (ii) elucidating the mechanisms employed by trehalose-degrading enzymes belonging to the glycoside hydrolases GH37, GH15, and GH65, and (iii) discussing the potential applications of these enzymes in different sectors. Notably, this review emphasizes the bacterial trehalose-degrading enzymes, specifically trehalases (GH37, GH15, and GH65) and trehalose phosphorylases (GH65), in both Gram-positive and Gram-negative bacteria, an aspect that has not been highlighted before.

## Introduction

Trehalose is a non-reducing disaccharide that because of its distinct physiochemical features, is synthesized by various organisms for survival strategies under extreme environments, which features enable it to protect cell integrity (Luyckx and Baudouin 2011). It is noteworthy that although the biosynthetic pathway for trehalose (OtsA/OtsB, TreY/TreZ, and TreS) is prevalent in many organisms (prokaryotic to eukaryotic forms), bacterial trehalose is not commonly found (Barraza and Sánchez 2013). This occurs because TreS is engaged in the degradation of trehalose, instead of its synthesis from trehalose to maltose, as proven through experiments with recombinant mycobacterial TreS (Wolf et al. 2003). There are various other ways in which trehalose can also be degraded (Caspi et al. 2016). Following enzymatic hydrolysis by trehalase, the disaccharide trehalose, comprising two α–glucose units, is broken down into an α–glucose molecule and a β–glucose molecule (Shukla et al. 2015). In comparison to bacterial trehalases, studies on insects and yeast trehalases have been more comprehensive. Trehalase plays a vital role in the metabolism of trehalose in both yeast and insects, providing energy, assisting in the reaction to stress, and taking part in a variety of physiological processes (Wyatt 1967). On the other hand, bacteria employ trehalose degradation pathways to obtain nutrients, respond to stress, regulate trehalose levels, store carbon and energy, and interact with host organisms. It is important to highlight that although certain bacteria engage in both trehalose degradation and synthesis, not all bacteria are involved in trehalose degradation. Six routes of trehalose degradation pathways have been reported in organisms depending on their subcellular locations (Avonce et al. 2006). It is crucial to emphasize that although different organisms may employ diverse processes to synthesize trehalose, trehalase stands as the exclusive enzyme that is accountable for the irreversible degradation of this trehalose in all organisms (Avonce et al. 2006). Trehalose that cannot undergo modification may be broken down by a hydrolyzing trehalase (EC 3.2.1.28), or it may be divided by trehalose phosphorylase (EC 2.4.1.64 and EC 2.4.1.231) (Shukla et al. 2018; Zhou et al. 2019; Caspi et al. 2020; Shrestha et al. 2022). At present, the CAZy (Carbohydrate-Active Enzyme) database (http://www.cazy.org/), categorizes glycoside hydrolases (GHs) into 180 protein families. Trehalase is a member of several GH families among them, including GH37, GH65, and GH15 (Lombard et al. 2014). While trehalase is the only enzyme in the GH37 CAZyme subfamily, certain acid trehalases and some phosphorylases belong to the GH65 CAZyme subfamily (Henrissat and Bairoch 1993; Carroll et al. 2007; Cantarel et al. 2009). Both families are GH–L or GH–G clan members and have the same traditional (α/α)6-barrel fold and inverted response mechanism (Kötzler et al. 2014).

This review provides an overview of the various pathways involved in the breakdown of trehalose and the enzymes that participate in these pathways. Additionally, we emphasize for the first time that the degradation of trehalose in bacteria is not solely dependent on trehalase enzymes; other enzymes, like trehalose phosphorylase, are also involved in trehalose degradation. Moreover, we wish to elucidate trehalose degradation pathways in bacteria.

## Mechanisms of bacterial trehalose-degradation pathways

Trehalose degradation pathways can vary depending on the microorganisms and the environmental conditions. In general, trehalose can be broken down into glucose by the action of the enzyme trehalase, which is present in many microorganisms. Glucose can then be further metabolized through glycolysis or other pathways, depending on the specific microorganism. Some microorganisms also have additional enzymes that can break down trehalose into other intermediates. For example, in *Escherichia coli* str. K-12, trehalose-6-phosphate hydrolase (TreC) may convert trehalose into glucose-6-phosphate and glucose, while cytoplasmic trehalase (TreF) can hydrolyze trehalose to produce glucose that can enter the periplasm (Elbein 1974; Ruhal et al. 2013).

**Fig. 1:**
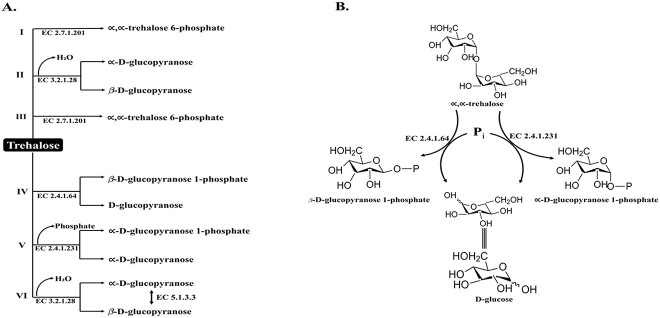
A) Six different trehalose degradation pathways (I, II, III, IV, V, and VI) are found in organisms. B) Reaction schematic of trehalose phosphorylase (EC 2.4.1.64 and EC 2.4.1.231) (Cornish-Bowden 2014; Shrestha et al. 2022).

In certain stressful conditions, such as exposure to high temperature or low water availability, trehalose can also be degraded through alternative pathways. For example, in some bacteria and yeast, trehalose can be broken down into glyceraldehyde-3-phosphate and dihydroxyacetone phosphate by the action of trehalose phosphorylase (Higuchi-Takeuchi et al. 2016). Overall, the degradation of trehalose in microorganisms can be a complex process that involves multiple enzymes and pathways and can be influenced by various environmental factors. The MetaCyc database has divided trehalose degradation pathways into six categories (Mahmud et al. 2010), and Fig. 1A summarizes this division.

### Trehalose degradation I

In this pathway, the breakdown of trehalose through the utilization of trehalose-6-phosphate is a common process in many bacteria, particularly Gram-negative bacteria like *Escherichia coli* str. K-12, under low osmolarity conditions. Trehalose-6-phosphate can breakdown trehalose in both low and high osmolarity conditions, but it can only synthesize the trehalose in high osmolarity conditions (Fig. 1A). In fact, *Escherichia coli* str. K-12 can thrive when trehalose serves as their sole carbon source. Under different osmolarity conditions, different pathways are switched on. In addition, under low osmolarity conditions, trehalose is not synthesized, and the only source is the external uptake of trehalose. Therefore, trehalose is imported into the cell using a phosphoenolpyruvate-dependent sugar phosphotransferase (PTS) system for trehalose, which is composed of the EIIA^Glc^ of the glucose-PTS, and a trehalose-specific EII^Tre^, encoded by the *treB* gene. The sugar PTS is a significant transport system for carbohydrates, responsible for both phosphorylating incoming sugar substrates and facilitating their movement across the cell membrane (Escalante et al. 2012). During transportation, trehalose undergoes phosphorylation, and enters the cytoplasm in the form of trehalose-6-phosphate. The trehalose-6-phosphate produced is subsequently degraded by trehalose-6-phosphate hydrolase, which is encoded by the *treC* gene. This hydrolysis reaction results in the formation of glucose and glucose-6-phosphate. The liberated glucose then undergoes additional phosphorylation by glucokinase, generating another molecule of glucose-6-phosphate, and then both of it enters the glycolysis pathway (Klein et al. 1995). Furthermore, glucose-6-phosphate serves as the initial stage in glucose metabolism, and forms a metabolic link between glycolysis, the pentose phosphate pathway, glycogen synthesis, de novo lipogenesis, and the hexosamine pathway (Rajas et al. 2019). Nevertheless, it has also been observed in certain Gram-positive bacteria, such as *Paenibacillus popilliae* and *Bacillus subtilis* (Bhumiratana et al. 1974; Karp and Caspi 2011).

### Trehalose degradation II

Trehalose, which is unmodifiable, may undergo degradation by a hydrolyzing enzyme called trehalase (EC 3.2.1.28), or it may be cleaved by trehalose phosphorylase (EC 2.4.1.64 and EC 2.4.1.231) (Fig. 1B) (Shukla et al. 2015). Two kinds of trehalase (periplasmic and cytoplasmic) can be found in bacteria depending on their subcellular location. Like, periplasmic trehalase (TreA) hydrolyzes exogenous trehalose when the osmotic pressure is high (Boos et al. 1987). The glucose-PTS then transports the resulting glucose molecules back into the cytoplasm (Styrvold and Strom 1991). Cytoplasmic trehalase (TreF), is another trehalase that is active when the osmolarity varies between high and low. TreF has a relatively low level of enzymatic activity, which is insufficient to prevent the synthesis of trehalose under conditions of high osmolarity. However, when conditions return to normal and trehalose synthesis cease, its activity rises to a level where it can degrade the trehalose that has accumulated (Horlacher et al. 1996). According to the CAZy database, cytoplasmic and periplasmic are categorized in the GH37 subfamily (Lombard et al. 2014).

### Trehalose degradation III

An inorganic phosphate-dependent enzyme called trehalose-6-phosphorylase is used by some species like *Lactococcus lactis*, that degrade phosphorylated trehalose (Andersson et al. 2001). In this case, trehalose enters the cell by a trehalose-PTS, where it is phosphorylated to form trehalose-6-phosphate. Trehalose-6-phosphorylase enzyme facilitates the reversible transformation of trehalose-6-phosphate into β-D-glucopyranose 1-phosphate and β-D-glucose 6-phosphate (Andersson et al. 2001). Both β-glucose 6-phosphate molecules then participate in the glycolysis pathway (Levander et al. 2001).

### Trehalose degradation IV

The main enzyme in this pathway is trehalose phosphorylase (EC 2.4.1.64), which catalyzes the reversible synthesis (and degradation) of trehalose from β-D-glucose-1-phosphate and D-glucose (Fig. 1). However, two different types of trehalose phosphorylase enzymes have been identified. One kind of enzyme is specific for the α-forms of D-glucose and D-glucose-1-phosphate, while the other enzyme is specific for the β-forms. Due to the distinct processes that the two forms catalyze, they were assigned different EC numbers 2.4.1.231 and 2.4.1.64 for α (retaining trehalose phosphorylase) and β (inverting phosphorylase) forms, respectively. When trehalose is broken down, the phosphorylase produces glucose-1-phosphate, which is then subsequently converted into glucose-6-phosphate by certain phosphoglucomutase (which can also be either α- or β-specific) (Aisaka and Masuda 1995; Aisaka et al. 1998). The β-specific trehalose phosphorylase enzymes have been found in various organisms, including bacteria like *Geobacillus setarothermophilus* (Aisaka et al. 1998), *Asanoa ferruginea* (Inoue et al. 2002a), *Thermoanaerobacter brockii*, *Plesiomonas* sp. (Inoue et al. 2002b), and *Bradyrhizobium japonicum* (Yoshida et al. 1997).

### Trehalose degradation V

Here also, trehalose phosphorylase is involved in the synthesis and breakdown of trehalose to D-glucose-1-phosphate and D-glucose (Fig. 1A). However, it is important to note that there is a distinction in the assigned EC numbers 2.4.1.231 (Fig. 1B) (Cornish-Bowden 2014). This enzyme is designed for α-specific reactions and is often found in fungi, although similar enzyme was also discovered in yeast *Pichia fermentans* (Eis and Nidetzky 1999). The retaining phosphorylases within this pathway utilize a two-step, double-displacement mechanism involving nucleophilic participation and the formation of a covalent glycosyl-enzyme intermediate (Fig. 1B) (Lairson et al. 2008). While this enzyme has been discovered in various fungi, its potential for use in synthesis remains unexplored (Kitamoto et al. 1998).

### Trehalose degradation VI

The GH37 (EC 3.2.1.28) family trehalase is responsible for converting α, α-trehalose into two glucose molecules while inverting the anomeric configuration. When there is high osmolarity, the PTS pathway for trehalose uptake is blocked. However, TreA allows the cell to still utilize trehalose (Gutierrez et al. 1989). TreA breaks down external trehalose into glucose molecules, which are then transported into the cytoplasm via the glucose-PTS (Giaever et al. 1988). Furthermore, in situations of high osmotic pressure, the bacteria produce a significant amount of trehalose as an osmoprotective measure. TreA plays a role in recycling any leaked trehalose from the cytoplasm into the periplasm, ensuring that the bacteria conserve this vital molecule (Styrvold and Strom 1991).

## Phylogeny of trehalase and trehalose phosphorylase

**Fig. 2:**
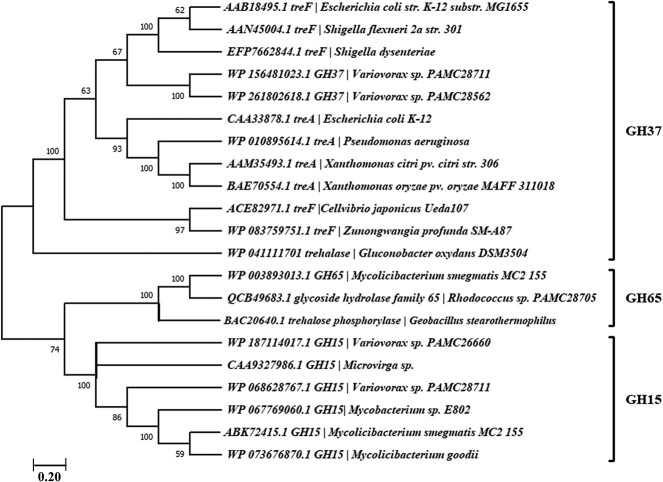
The related enzyme sequences were analyzed to determine their evolutionary relationships. The multiple sequence alignment was performed using MUSCLE (Edgar 2004). A phylogenetic tree was created using the neighbor joining method in MEGA X (Saitou and Nei 1987). The nodes of the tree bootstrap values were obtained from 1,000 replicates. Additionally, the accession number, enzyme name, and organism name are provided alongside the tree. The reference strains were used for their respective trehalose-degrading genes as *Escherichia coli str. K-12 substr. MG1655* (GH37), *Geobacillus stearothermophilus* (GH65) and *Mycolicibacterium smegmatis MC2 155* (GH15).

To classify the GH family for trehalose-degrading enzymes, we conducted a phylogenetic tree analysis of the characterized and predicted enzymes using the neighbor-joining method (Kumar et al. 2018) with trehalose-degrading strains that have been reported and predicted previously. We took reference strains, *Escherichia coli* str. K-12 substr. MG1655, *Geobacillus stearothermophilus*, and *Mycolicibacterium smegmatis* MC2 155 for GH37, GH65, and GH15, respectively as trehalose-degrading enzymes that have previously been reported for their respective GH families (Fig. 2). Interestingly, this revealed that GH15 is present in both Gram-positive and Gram-negative bacteria, but GH65 is only found in Gram-positive bacteria. The GH37 was also found to be more prevalent in Gram-negative bacteria only (Fig. 2). Notably, it is also observed that GH37 trehalase possesses two kinds of trehalase based on their location i.e. cytoplasmic TreF, and periplasmic TreA. As previously mentioned, when the cell experiences low osmolarity conditions, TreF is responsible for metabolizing cytosolic trehalose. On the other hand, when the osmolarity is high, periplasmic TreA is involved in the utilization of extracellular trehalose (Moon et al. 2016; Zhang et al. 2022). According to the analysis conducted by Sakaguchi et al. (2015), the GH15 domain is believed to have evolved after the GH37 and GH65 domains. It is currently found in bacteria, as well as a limited number of archaea. In previous publications, we have presented similar findings concerning our polar bacteria species, *Variovorax* sp. PAMC28711 (Shrestha et al. 2022a). This species is known to possess two types of trehalases, namely GH15 and GH37. Additionally, another strain called *Shigella* sp. PAMC28760 (Shrestha et al. 2022b) was found to have two GH37 trehalase genes, specifically *treA* located in the periplasm, and *treF* located in the cytoplasm.

In 2019, Tellis et al. found that eukaryotes have a trehalase presence of 51.9%, compared to bacteria’s prevalence of 47.6%, and archaea’s presence of only 0.265%. Fungi make up 21.4% of eukaryotes, while metazoans make up 21.2%. Despite the diversity and wide range of distribution of trehalase, its molecular function is limited, and mostly regulates specific groups of biological activities. The classification of trehalases containing trehalase domains from the GH37 family and GH65 family into different clades suggests diverse ancestral origins (Tellis et al. 2019). Trehalase is a crucial component of carbohydrate metabolism, because based on a gene ontology analysis, it performs molecular tasks like cleaving trehalose’s glycosyl bonds, and perhaps taking part in ion or metal binding (Ashburner et al. 2000). To substantiate metal binding, Franco et al. (2003) found the physical binding of Ca^2+^ to neutral trehalase of fission yeast, *Schizosaccharomycs prombe* consists of an EF-motif that is similar to that shown by many Ca^2+^-binding proteins. This binding of Ca^2+^ ions is the requirement for correct enzyme folding to its active form for proper functionality. Trehalose hydrolysis is essential for energy-intensive activities in biological processes and has a substantial impact on how cells react to stress or stimuli. A more comprehensive investigation into trehalase’s cellular components reveals that it is mostly present in the cytoplasm at (60%–70%), while a smaller amount is connected to the cell wall or found extracellularly beside acidic trehalase from some eukaryotes (Ashburner et al. 2000). Trehalase’s biological function can vary depending on variances in its sequence and structure. Therefore, studying the evolutionary characteristics of the enzyme is essential to understanding how it has changed over time.

## Trehalose degradation pathways in gram-positive and gram-negative bacteria

**Fig. 3:**
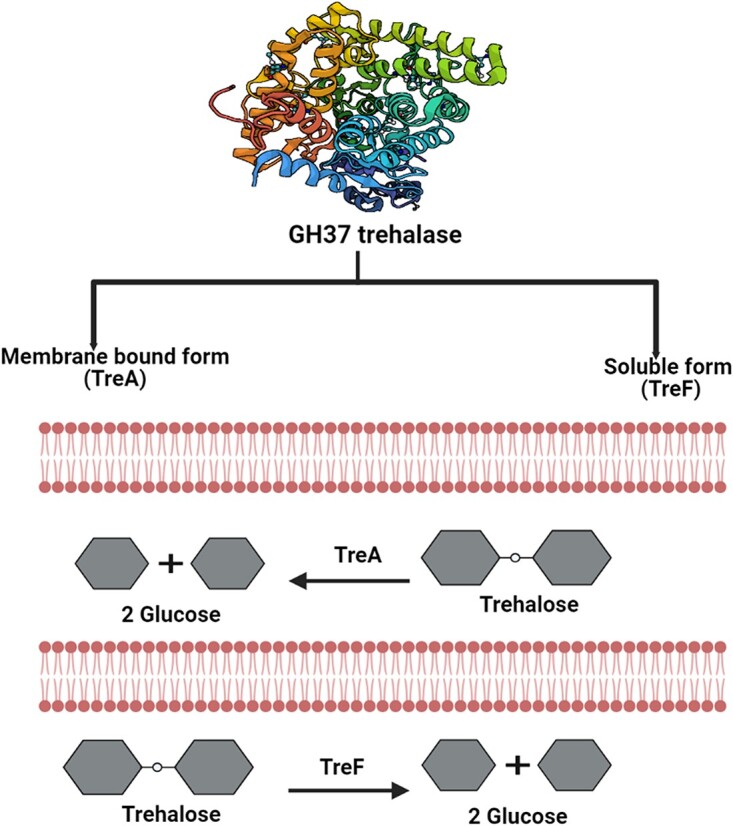
Bacterial trehalase. Occurrence of soluble and membrane-bound forms of GH37 trehalase in bacteria.

Trehalose degradation mechanisms vary between Gram-positive and Gram-negative bacteria (Kitamoto et al. 1988; Han et al. 2003). Trehalase likely originated in bacteria and spread to other organisms through horizontal gene transfer (Collins et al. 2018). There are two types of GH37 trehalases, TreA and TreF, which differ in subcellular location, function, and distribution (Fig. 3). In *Escherichia coli* str. K-12, both periplasmic (TreA) and cytoplasmic (TreF) trehalases have been observed (Elbein et al. 2003). While both genes are affected by osmolarity, *treA* exhibits significantly higher enzymatic capacity, even under low osmolarity conditions, compared to *treF* (Horlacher et al. 1996). TreF has been reported to be found in various organisms, including yeast (*Saccharomyces cerevisiae*) and insects, where it plays critical roles in energy production, stress response, and developmental processes (Boos et al. 1987; Argüelles 2000), whereas periplasmic trehalase is commonly found in certain bacteria, such as *E. coli* str. K-12 and other Gram-negative bacteria, where it assists in trehalose utilization and nutrient acquisition (Horlacher et al. 1996). Though both enzymes are dependent on osmolarity, their osmoregulation does not solely rely on the stationary-phase sigma factor, *rpoS* (Repoila and Gutierrez 1991). The discovery of GH15 trehalases occurred relatively late, and only a few of them from archaea, such as *Thermoplasma volcanium*, *Thermoplasma acidophilum*, and *Sulfolobus acidocaldarius*, have been biochemically characterized (Moon et al. 2016; Zhang et al. 2022). There are also limited reports on the characterization of GH15 bacterial trehalases, except for a recently isolated trehalase from the strictly aerobic bacterium *Microvirga* sp. MC18. GH15 trehalase was identified and first reported in 2007 in *Mycobacterium smegmatis* and *Mycobacterium tuberculosis* (Carroll et al. 2007). The spores of *Streptomyces hygroscopicus* were discovered to have high levels of trehalose, but low levels of the enzyme trehalase. Trehalase activity began to increase during spore germination before any noticeable increase in cell mass occurred. After approximately 25 h, the enzyme’s activity peaked at nearly 20 times its initial level; meanwhile over the same period, intracellular trehalose levels decreased substantially. These observations imply that the trehalase plays a crucial role in the early stages of spore germination (Elbein 1974). Some bacteria use exogenous trehalose as a carbon and energy source instead of synthesizing it (Kandror et al. 2002). *B. subtilis* and *Clostridium difficile* are two prominent examples of these microorganisms (Helfert et al. 1995; Schöck and Dahl 1996; Schönert et al. 2006). Moreover, there is another transporting system called LpqY-Sugabc, which is responsible for transporting periplasmic trehalose. This system has been observed in *Mycobacteria smegmatis ΔsugC* strains and is involved in moving liberated from the breakdown of trehalose dimycolate. Trehalose dimycolate hydrolase facilitates this process, especially under carbon-limited conditions (Ojha et al. 2010; Holmes et al. 2019; Pohane et al. 2021). Once trehalose internalized, some portion of it potentially converts into trehalose monomycolate (trehalose-containing glycoconjugates). This transformation is supported by the detection of DBCO-Cy5 labeled trehalose in *M. smegmatis* and *M. tuberculosis* (Swarts et al. 2012; Pohane et al. 2021). This suggests a form of trehalose recycling, which helps maintain trehalose monomycolate levels and serves as a substrate for trehalose-degrading enzymes.

**Fig. 4:**
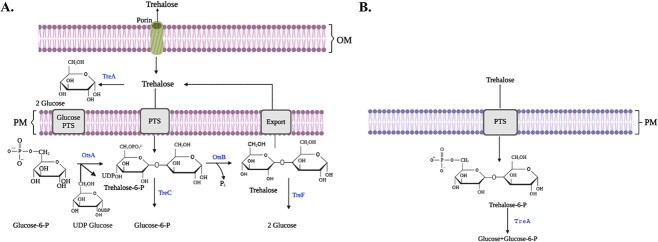
Representative trehalose degradation pathways in A) gram-negative bacteria (*Escherichia coli*), and B) gram-positive bacteria (*Bacillus subtilis* and *Clostridium difficile*) (Kalera et al. 2020). Many other bacteria also possess similar trehalose-degradation processes. OM, outer membrane; PM, periplasmic membrane; PTS, phosphotransferase system; and P, phosphate.

There are variations among the structural components of Gram-positive and Gram-negative bacterial cell walls. Gram-positive bacteria, unlike Gram-negative bacteria, don’t normally have an outer membrane, porins, or lipopolysaccharides (Fig. 4). Notably, porins play a crucial role in transportation of nutrients within the cell wall and bacterial physiology. Porins might not be directly involved in the enzymatic processes that break down trehalose, but they do make it easier for nutrients, ions, and other small molecules to diffuse. This diffusion is crucial for the overall metabolic processes of bacteria, including the utilization of trehalose as an energy source (Kalera et al. 2020). It is significant to note that there has not been much research on the precise role of porins in trehalose breakdown pathways, and their function may vary depending on the bacterial species and environmental conditions. To fully comprehend the significance of porin involvement in trehalose metabolism, additional research is required. Additionally, it has been reported that some of the bacterial species, such as *Burkholderia pseudomallei* and *Xanthomonas) citri* subsp. *citri*) possess trehalase that plays a crucial role in stress adaptation and virulence (Vanaporn et al. 2017; Alexandrino et al. 2016).

## Architecture of trehalose-degrading enzymes

Trehalase and trehalose phosphorylase are two enzymes that aid in the degradation of trehalose. These enzymes are members of the glycoside hydrolase GH37, GH15, and GH65. GH37 and GH15 include trehalase, while GH65 contains both trehalase and trehalose phosphorylase. Both the 15 and the 65 trehalase families have a bacterial and fungal origin and share a glutamic acid (Glu) residue as a proton donor. However, the nucleophilic groups of the two families are different from one another. In the 15 family, the nucleophile is Glu, whereas in the 65 family, it is water. On the other hand, Glu serves as the nucleophile, while aspartic acid (Asp) serves as the proton donor for the catalytic activity of trehalases belonging to the 37 family (Silva et al. 2010). GHs employ one of two catalytic mechanisms: either retaining, or inverting. Trehalase is a glycosidase with an inverting mechanism that yields products with stereochemistry opposite to the substrate (Davies and Henrissat 1995; Henrissat et al. 1995; Zechel and Withers 2000). On the other hand, trehalose phosphorylase can act as a retaining or an inverting glycosidase (Fig. 1B). The CAZy database (https://www.cazy.org/) has classified the GH family into distinct “clans” based on their presumed shared evolutionary origins. Within this classification, the GH37 enzymes were grouped under clan GH-G, while the GH65 and GH15 enzymes were assigned to clan GH-L. Although clans GH-G and GH-L exhibit limited sequence homology, this observation holds significant importance (Xu 2020).

**Fig. 5:**
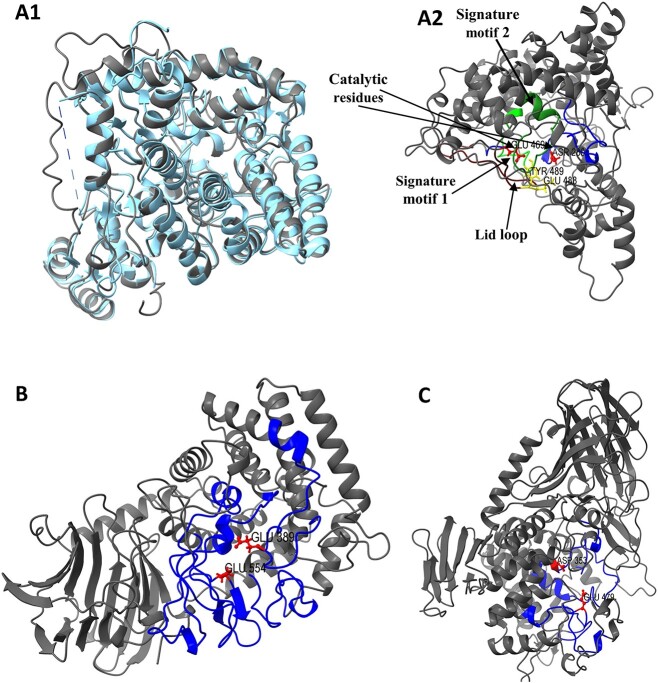
Structure of trehalose-degrading proteins. A1). Structural alignment using the Chimerax matchmaker tool of crystallographic solved GH37 protein (PDB No. 2JF4) and proposed GH37 trehalase (RMSD is 0.674, while sequence alignment score is 1530.4). A2). GH37 trehalase with proposed signature motif 1 and motif 2, catalytic residues, and the lid loop. B) GH15 trehalase, and C) GH65 trehalose phosphorylase, with proposed catalytic residues, respectively.

The glycoside hydrolase family GH37 only has one enzyme, trehalase (EC 3.2.1.28), whereas the GH65 family consists of many enzymes that are responsible for degrading polysaccharides, and two of these enzymes are trehalase (EC 3.2.1.28) and trehalose phosphorylase (EC 2.4.1.64/EC 2.4.1.231) that are involved in the degradation of trehalose. Likewise, compared to the GH37 family, the GH15 family also has many enzymes besides one trehalose-degrading enzyme trehalase (EC 3.2.1.28). The (α/α)6-barrel catalytic domains can be seen in GH37, GH65, and GH15 enzymes. Although crystal structures have only been resolved for GH37 trehalases, it is assumed that GH15 and GH65 trehalases share common catalytic domains with (α/α)6-barrel structures (Table 1) (Xu 2020). GH37 trehalase contains two catalytic residues, Asp and Glu, while GH65 and GH15 trehalases have Asp and Glu residues, which could participate in a shared inverting catalytic mechanism (Adhav et al. 2019). The basic structures of the GH37 enzymes revealed the presence of two well-known trehalase signature motifs, namely motif 1 (PGGRFXEXY[G/Y] WD[S/T] Y), and motif 2 (QWD[Y/F] P[N/Y] [G/A] W[P/A] P). On the other hand, these patterns are absent from the GH65 and GH15 trehalases. Along with the two well-known trehalase signature motifs (motifs 1 and 2), three additional conserved regions, designated as motifs 3 (N[A/G] XRXYYXXRSQPP), 4 (SGXD[T/F] [S/T] [S/T/Y] R[F/L/W]), and 5 (EK[Y/F] D), have been proposed for the GH37 enzyme catalytic domains. The two catalytic residues mentioned above are present in motifs 4 and 5. In motif 5, lid loop sections are also visible, and they might be crucial for substrate identification (Sakaguchi 2020). Figure 5 shows the predicted structures of GH65, GH15, and GH37 trehalases structures that have been described with their catalytic residues, signature motifs, and catalytic and conserved regions based on already reported amino acids. GH37 trehalase is distributed diversely, while GH65 trehalase has exclusively been found in fungi and yeasts, with activity observed under acidic conditions. GH15 trehalase has been reported solely in archaea and bacteria.

**Table 1:** Classification of trehalose-degrading enzymes.

Family	Enzyme	EC number	Clan	Mechanism	Linkage	3D structure status	Donor	Acceptor	Product	References
GH65	α, α- trehalase	3.2.1.28	GH-L	Inverting a	α-glucosidic	(α/α)_6_ barrel	D-Glu/D-Glu-6-P	Glu/β-Glu1P	α- and β- glucose	Sun and You (2021)
	α, α- trehalose phosphorylase	2.4.1.64/2.4.1.231	GH-L	Inverting a	α- glucosidic	(α/α)_6_ barrel	α-Glucosides/β-Glu 1-P	Glu/β-Glu1P	β-D-Glu 1-P/α-D-Glu1-P	Lairson et al. (2008)
GH37	α, α- trehalase	3.2.1.28	GH-G	Inverting a	α-glucosidic *O*-	(α/α)_6_ barrel	UDP-Glu/Glu-6-P	Glu-6-P	α- and β- D-glucose	Gibson et al. (2007)
GH15	α, α- trehalase	3.2.1.28	GH-L	Inverting a	α, α- (1,1)-glucosidic	(α/α)_6_ barrel	D-Glu/D-Glu-6-P	Glu/β-Glu1P	α-and β-D-glucose	Sakaguchi (2020)

## Application of trehalose-degrading enzymes and their future perspectives

### Trehalase

**Fig. 6:**
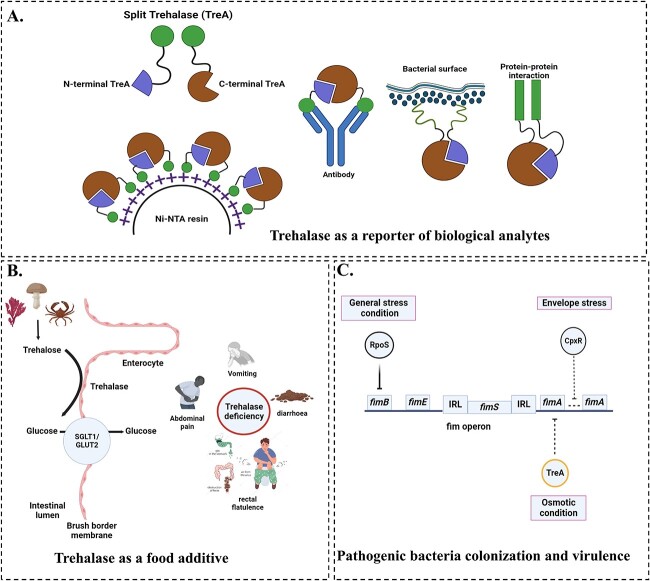
Role of trehalase. A) Trehalase as a reporter on biological analytes. B) Trehalase as a food additive. C) Pathogenic bacteria colonization and virulence. Schematics were generated using BioRender.com.

Although trehalase (EC 3.2.1.28) exists in both periplasmic and cytoplasmic forms in bacteria, periplasmic trehalase has been extensively studied and applied in various ways. Notably, it is used in a split reporter enzyme, where it degrades trehalose into two glucose molecules that can easily be measured using a commercial glucometer. Concisely, the split TreA fragments comprise defined N-terminal and C-terminal regions with His tag at N- or C-terminal that are individually generated, expressed, and purified. The successful split TreA complementation was achieved exclusively upon immobilizing all fragments onto Ni-resin with distinct protein binding domains, followed by co-incubation with trehalose and specific proteins, resulting in glucose production. This technology can detect a broad range of analytes, including antibodies, bacteria, protein–protein interactions, and protein aggregation (Drikic and De Buck 2018). Mukherjee and De Buck introduced a novel assay for Bovine Leukemia Virus (BLV) using p24 antigen specificity. This assay has demonstrated great potential as a simple and rapid diagnosis of BLV infection. The study explored the application of split enzyme sensor diagnostic technology to identify immunoglobulins and antigen-specific antibodies using *Escherichia coli* str. K-12 periplasmic TreA (Mukherjee and De Buck 2021) (Fig. 6A). Before starting field testing, more analysis and modifications of this assay are required. This may involve testing blinded or randomized field samples, as well as using pre-assembled freeze-dried reagents and a readily available handheld glucometer to detect the output signal. Additional modifications to this methodology could lead to the development of a cost-effective diagnostic approach that can be easily customized for pathogen-specific testing.

The digestive enzyme α-glucosidase trehalase, located in the intestinal brush border, breaks down trehalose present in mushrooms, yeast, and other single-cell food sources (Murray et al. 2000). Trehalase deficiency is rare, except in Greenland Inuit populations, where approximately (10%–15%) lack this enzyme. However, certain Caucasians may experience mild gastrointestinal discomfort when consuming trehalose-rich mushrooms (Arola et al. 1999). Therefore, introducing commercial trehalase supplements in dried foods could potentially alleviate such sensitivity cases and promote healing (Fig. 6B).

The inhibition of type 1 fimbriae expression by periplasmic trehalase (TreA), in addition to other stress conditions, has been linked to osmotic stress. The deletion of *treA* gene for extraintestinal pathogenic *Escherichia coli* MT78 leads to an increase in osmotic resistance to urea, but the inhibition of type 1 fimbriae expression, as observed in reduced colonization in murine UTI model uroepithelium (Pavanelo et al. 2018). Vanaporn et al. (2017) also reported that *treA* mutant has a reduced ability to survive in macrophages after it is attenuated in both *Galleria mellonella* (larva), and mouse infection models (Vanaporn et al. 2017). Proteins associated with the synthesis of trehalose have been identified as potential targets for new drugs. This metabolic pathway is not present in mammalian cells, and the enzymes involved in this pathway exhibit high specificity. This finding indicates that trehalase contributes to the virulence of *Burkholderia pseudomallei*, highlighting the importance of exploring this pathway as a potential drug target for the treatment of melioidosis (Fig. 6C).

### Trehalose and its analogues synthesis via trehalose phosphorylase

**Fig. 7:**
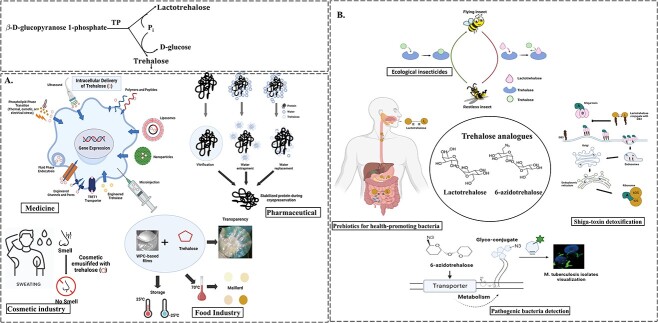
Role of trehalase phosphorylase. A) Trehalose phosphorylase acts in the synthesis of trehalose, which has further application. B) Trehalose analogues and their roles in different areas. Schematics were generated using BioRender.com.

As mentioned earlier, trehalose phosphorylase acts in reversible reactions depending on the environment and microorganisms. Trehalose phosphorylase is being explored for its potential in both trehalose synthesis and downstream applications. Trehalose can be incorporated as a crucial component in cosmetic products to combat body odor caused by the production of unsaturated aldehydes (such as 2-nonenal and 2-octenal) associated with ageing. In a particular study, seniors (aged >55 years) had their bodies sprayed with a 2% trehalose solution after showering, and analysis revealed a reduction of approximately 70% in odor from their shirts after a 20 h period (Fig. 7A) (Higashiyama 2002; Oku et al. 2002). Additionally, trehalose has been widely utilized in various pharmaceutical formulations as a stabilizer. This is primarily achieved through three mechanisms: reducing the movement of biomacromolecules by either sequestering or replacing water, or by inhibiting solvent crystallization by creating a glassy matrix around unstable biomolecules (Vinciguerra et al. 2022). For example, when it comes to stabilizing pyrophosphatase and glucose-6-phosphate dehydrogenase against heat, trehalose has shown to be approximately twice as effective as an equivalent amount of sucrose and maltose. In comparison, trehalose has proven to be a superior protectant for liposomes compared to sucrose when subjected to lyophilization, followed by storage or heating conditions. Cryopreservation techniques are widely employed in stem cell technology and regenerative medicine for cell storage. Trehalose is used as an extracellular protective agent for cryopreservation, although it falls short in comparison to the widely used cryoprotective agent DMSO, due to its inability to permeate the mammalian cell membrane (Crowe et al. 2005). One of the intriguing methods investigated for introducing trehalose into mammalian cells for their storage at cryogenic and ambient temperatures is the use of nanoparticles (Rao et al. 2015). Rao et al. (2015) developed thermally responsive nanoparticles containing genipin, which effectively delivered trehalose into human adipose-derived stem cells and allowed for their cryopreservation (Fig. 7A). The release of trehalose from the nano-capsules occurred in response to a pH change at 37 °C (Rao et al. 2015). In the realm of food applications, trehalose shows promise as a viable alternative to protein-based film formulations. This is primarily attributed to its ability to maintain stability under both low and high temperatures, as well as its non-participation in the Maillard reaction. When incorporated into whey protein film, the addition of trehalose effectively prevents browning, and retains sufficient transparency, making it suitable for see-through packaging purposes (Pérez et al. 2016).

It is believed that the synthesis of trehalose analogs (Fig. 7B) using carbohydrates other than glucose offers distinct metabolic advantages. For example, lactotrehalose (α-D-galactopyranosyl α-D-glucopyranoside), in which the glucose unit is replaced by galactose, functions not as a substrate for intestinal trehalase, but as a competitive inhibitor of the same enzyme (Lin-Goerke et al. 1997). Additionally, lactotrehalose remains unhydrolyzed in the intestine and reaches the colon, where it may exert prebiotic effects. Certain di-, tri-, or tetrasaccharides containing α-galactosyl, α-glucosyl, and α-fructose residues are known to selectively stimulate the proliferation of bifidobacterial, which promotes good health (Minami et al. 1985). Recently, lactotrehalose has been proposed as a molecular mimic of globosyl (Gb) disaccharide (Dohi et al. 2002). For example, lactotrehalose conjugated to Gb3 ceramide has been suggested to detoxify and neutralize Shiga toxin, a major pathogenic component in enterohemorrhagic fever (Fig. 7B) (Nataro and Kaper 1998; Miyachi et al. 2009).

The inhibition of trehalase presents an interesting prospect for developing environmentally friendly insecticides, given that trehalose serves as a vital energy source for insect flight (Gibson et al. 2007). In addition, by hindering glucose transport and upregulating insulin signal regulator genes, a trehalase-resistant analog has the potential to be developed as a next-generation fasting mimetic drug for treating conditions such as diabetes and nonalcoholic fatty liver disease. This was observed through the decreased abundance of *Clostridium difficile* strain CD0204 (Zhang et al. 2020). Trehalose analogs also possess intriguing qualities for the healthcare sector. For example, trehalose is an essential metabolite for *Mycobacteria*, and its azido-derivative can serve as a tool for imaging and identifying *Mycobacterium tuberculosis*, the causative agent of tuberculosis (Urbanek et al. 2014). Similarly, trehalose derivates, such as validamycin A (R = β-D-glucopyranosyl) and validamycin A (R = H) have shown a half maximal inhibitory concentration of 1.8 × 10^−3^ mM and 1.1 × 10^−5^ mM, respectively, towards *Mycobacterium smegmatis* trehalase (Dhaene et al. 2020). Despite their attractiveness as a target, to date there are no trehalase inhibitors that have been developed as commercial bactericides or insecticides (Matassini et al. 2020).

## Conclusion and outlook

In our review, we have analyzed the trehalose-degrading enzymes that have been reported before. Upon further analysis, it was observed that GH15 trehalase is present in both Gram-positive and Gram-negative bacteria, whereas GH37 trehalase is predominantly found in Gram-negative bacteria. In the case of GH65 trehalose phosphorylase, it has only been reported in Gram-positive bacteria, and it was shown in the phylogenetic tree analysis as well. Interestingly, the analysis of cell walls in Gram-positive and Gram-negative bacteria revealed a potential role of porin in trehalose metabolism, which requires further investigation and additional research. Trehalase and trehalose phosphorylase from GH65 were further analyzed, and it was discovered that only trehalose phosphorylase is responsible for bacterial trehalose degradation. The GH65 trehalose phosphorylase is further categorized into α and β form based on retaining (α-form/EC 2.4.1.231), and inverting (β-form/EC 2.4.1.64), configuration, respectively. Notably, we found that inverting trehalose phosphorylase (EC 2.4.1.64) is present in both bacteria and fungi, while retaining trehalose phosphorylase (EC 2.4.1.231) has been reported in bacteria thus far. Additionally, it was discovered that inverting phosphorylase is also involved in trehalose synthesis alongside catalysis. However, no trehalose synthesis pathway has yet been identified for retaining phosphorylase in bacteria.

Trehalose-degrading enzymes including trehalase and trehalose phosphorylase have been reported in previous analyses, highlighting their distinct catalytic properties and widespread utilization in a wide range of applications. However, not much progress has been achieved in comprehending the crystal structures of bacterial trehalases and trehalose phosphorylase, apart from the crystallization of periplasmic TreA from *Escherichia coli* str. K-12. Furthermore, due to their low production efficiency and suboptimal catalytic properties, the industrial application and commercialization of trehalase and trehalose phosphorylase have been limited, posing challenges to realizing their full potential. In this review, we have sought to provide valuable insights into the variation in trehalose-degrading enzymes; not only do trehalases belong to glycoside hydrolase GH37, GH15, and GH65, trehalose phosphorylase, which belongs to GH65, is an additional enzyme that is responsible for trehalose degradation. Therefore, we believe that elucidating the trehalose utilization mechanism between Gram-positive and Gram-negative bacteria will be helpful to enhance the production and commercialization of these enzymes for various biotechnological, bioremediation, and industrial applications.
